# Mapping the Hidden Hazards: Community-Led Spatial Data Collection of Street-Level Environmental Stressors in a Degraded, Urban Watershed

**DOI:** 10.3390/ijerph15040825

**Published:** 2018-04-22

**Authors:** Na’Taki Osborne Jelks, Timothy L. Hawthorne, Dajun Dai, Christina H. Fuller, Christine Stauber

**Affiliations:** 1 Department of Public Health, Agnes Scott College, 141 E. College Avenue, Decatur, GA 30030, USA; 2 Department of Sociology, University of Central Florida, 4000 Central Florida Blvd., Phillips Hall, Room 403-P, Orlando, FL 32816, USA; timothy.hawthorne@ucf.edu; 3 Department of Geosciences, Georgia State University, 24 Peachtree Center Ave. NE, Atlanta, GA 30302, USA; ddai@gsu.edu; 4 Division of Environmental Health, Georgia State University School of Public Health, P.O. Box 3995, Atlanta, GA 30302, USA; cfuller@gsu.edu (C.H.F.); cstauber@gsu.edu (C.S.)

**Keywords:** participatory mapping, community GIS, participatory GIS, community-based participatory research (CBPR), Proctor Creek, Atlanta, GA

## Abstract

We utilized a participatory mapping approach to collect point locations, photographs, and descriptive data about select built environment stressors identified and prioritized by community residents living in the Proctor Creek Watershed, a degraded, urban watershed in Northwest Atlanta, Georgia. Residents (watershed researchers) used an indicator identification framework to select three watershed stressors that influence urban livability: standing water, illegal dumping on land and in surface water, and faulty stormwater infrastructure. Through a community–university partnership and using Geographic Information Systems and digital mapping tools, watershed researchers and university students designed a mobile application (app) that enabled them to collect data associated with these stressors to create a spatial narrative, informed by local community knowledge, that offers visual documentation and representation of community conditions that negatively influence the environment, health, and quality of life in urban areas. By elevating the local knowledge and lived experience of community residents and codeveloping a relevant data collection tool, community residents generated fine-grained, street-level, actionable data. This process helped to fill gaps in publicly available datasets about environmental hazards in their watershed and helped residents initiate solution-oriented dialogue with government officials to address problem areas. We demonstrate that community-based knowledge can contribute to and extend scientific inquiry, as well as help communities to advance environmental justice and leverage opportunities for remediation and policy change.

## 1. Introduction

Both natural and built environments contain environmental hazards and stressors such as air and water pollutants, solid and hazardous wastes, disease vectors, and stormwater that may negatively impact urban communities. The existence of these hazards and stressors is often coupled with inequitable distribution of exposures, risk, and vulnerabilities [1,2,3]. Urban settings—defined herein as areas within cities—therefore, pose special challenges to addressing population health and heath disparities [4,5,6,7]. 

The Framework for Urban Health posits that the health of urban populations is a function of urban living conditions and municipal-level determinants, as well as national and global social, economic, and political trends [8]. Due to the direct influence that urban living conditions have on the health of urban populations, this conceptual model suggests that they are the most feasible determinant to modify and that seeking to make “specific and targeted changes” in these conditions should be prioritized to improve the health of urban populations [8]. 

The built environment is inextricably linked to urban living conditions. Exploring the influence of the built environment on the health of urban populations helps to broaden understanding of the environmental health challenges in cities, as well as identify opportunities to make tangible built environment modifications to promote health and improve quality of life [2,9]. The existence and quality of municipal services such as sanitation, drainage, infrastructure maintenance, garbage collection, and access to safe drinking water shape the quality of the physical environment in urban areas. Studies that examine such environmental health challenges through a regulation and enforcement lens tend to support policy-level changes as measures to enhance health and well-being [9,10,11,12,13,14,15].

Citizen science is widely used to engage lay citizens in making ecological observations about the natural world [16,17,18]. In recent years, citizen science and other participatory approaches have also been utilized to conduct air and water quality monitoring and address a wide range of health and environmental justice challenges in community settings [19]. Few published studies, however, focus on built environment stressors, thereby presenting challenges with identifying evidence-based practice aimed at improving urban living conditions to promote health. As noted by Northridge et al., the theory that links built environment conditions to health and well-being has not been adequately supported with the empirical evidence needed to influence planning and policy changes [4]. 

Participatory mapping approaches apply citizen science principles and draw upon the fields of community mapping and Public Participatory Geographic Information Systems (PPGIS). Community mapping does not require professional mapping expertise. It is led by members of a community who use local knowledge to inform dialogue about particular spaces and the environmental, political, economic, and social conditions that shape them [20,21]. PPGIS is an approach through which GIS practitioners attempt to make GIS more accessible to members of the public and provide vehicles through which citizens are empowered to influence spatial decision making [22,23,24]. Through community-based participatory research and other community–academic partnerships, community knowledge can be joined with technical mapping expertise to create alternative community narratives that can influence investment of resources and urban policy and practice to improve environmental quality and promote health.

According to Calisaya, GIS can be powerful because of “…its ability to create visual images of the world based on scientific information, to unveil previously hidden natural and social landscapes with an authority of science” [25] (p. 15). Participatory approaches such as photovoice use photographs to raise awareness about critical community issues and advance policy change [26,27,28]. Pairing visual evidence with traditional analytical research methods, such as the use of GIS, makes research processes more accessible to and useful for citizens in crafting compelling community narratives that can be presented to fellow residents and decision makers and used as the basis for remedial action and better environmental management. Documenting community conditions both spatially and visually can assist community residents in influencing place-based decision making.

In the context of a degraded, urban landscape, using Atlanta, Georgia’s Proctor Creek Watershed as a focal area, this article asks the following question: how can citizens use their own knowledge and lived experience to document the existence of street-level, environmental hazards in their community? We answer this question through the process and findings of a collaborative community–university partnership, forged to elevate Proctor Creek Watershed residents’ knowledge of street-level environmental hazards, through creation of a novel, mobile application (app) and collection and analysis of spatial and visual data. Resident knowledge was leveraged to advance meaningful engagement in community decision making that achieves environmental justice and policy change.

## 2. Methodology 

### 2.1. Study Area 

The Proctor Creek Watershed is a 16 m^2^ watershed located in Northwest Atlanta, Georgia. The watershed is home to 38 neighborhoods, the historic homes of civil rights leaders such as Dr. Martin Luther King Jr., and more than 90,000 residents; the majority of whom are African American and experience social and economic disparities [29,30,31,32,33]. Although the watershed is home to several brownfield sites, a closed landfill, and some of the city’s lowest income populations, and most historically underserved neighborhoods [34,35,36,37,38,39], environmental, economic, and social challenges therein are not well documented in the literature. Recent peer-reviewed published literature draws connections between watersheds and human health and well-being. Specifically, the governance and management of land and water is being explored for its influence on these factors [40,41,42].

After decades of public disinvestment and neglect, watershed residents are faced with multiple environmental challenges that may pose health risks including: illegal dumping, impaired water quality, aging and polluting sewer infrastructure (combined sewer overflow system), pervasive flooding, and elevated risk for West Nile virus infection [29,30,31,32,33]. Proctor Creek and its tributaries flow through residential neighborhoods (including residential lots), public parks, and school grounds. Community meetings with watershed residents have also revealed anecdotal accounts of fishing in the stream for the purpose of consumption. Recently, Proctor Creek was designated a priority area for investment through the Urban Waters Federal Partnership, and this has resulted in increased interest in the area. Sites in the partnership benefit from the involvement of multiple federal agencies that work to leverage collective staff and financial resources, coordinate monetary investments, and engage with local partners to improve environmental quality and economic development opportunities [33,43,44].

### 2.2. Participant Recruitment and Selection

Study participants were identified for both the research described herein and a related Photovoice study, from September to October 2014, through use of recruitment flyers posted in community parks, recreation centers, and health clinics, as well as through face-to-face contact at community association and Neighborhood Planning Unit meetings and communities of faith within the Proctor Creek Watershed. The City of Atlanta is divided into 25 Neighborhood Planning Units, or NPUs. Each NPU has a citizen advisory council responsible for making recommendations to the Mayor and City Council on matters of zoning, land use, and a range of other social and economic determinants that influence health and quality of life. 

Early recruits were engaged to help identify other participants through snowball sampling to meet the desired sample size (*n* = 10) and to ensure representation from the majority of the six primary NPUs that comprise the watershed. Participants were required to be at least 18 years of age and reside in the Proctor Creek Watershed. Minors and those who do not live in the study area were excluded. In this article, we describe the participatory mapping study, however, participants were engaged in both this one and the aforementioned Photovoice study, in which Proctor Creek community assets and strengths as well as concerns and challenges were identified.

### 2.3. Community-Driven Research Agenda

This research, a part of a larger collaborative study co-designed and co-led by residents of the Proctor Creek Watershed, was conducted over a 5-month period and commenced with the identification of indicators representing street-level environmental hazards by 10 Proctor Creek Watershed adult residents (ages 29–66) who had lived in the watershed for between 8 and 66 years. These residents’ levels of educational attainment ranged from not having a high school diploma to being college degree recipients. Their significant local knowledge about Proctor Creek; lived experience in the watershed; and concern about hazards, they deemed to have a negative effect of the livability of their communities, were drivers of this research. 

Participants were compensated for their contributions as co-researchers to the study and are referred to as watershed researchers hereafter. The watershed researchers developed the aforementioned indicators in response to the following questions: (1) What contamination and pollution is in the Proctor Creek Watershed? (2) What potential human health impacts are there from this contamination and pollution? and (3) What actions can be taken and/or proposed to address these environmental and human health hazards? In beginning their research, the watershed researchers reviewed, compared, and contrasted existing data, including both publicly available “expert” data obtained from U.S. Environmental Protection Agency databases and data that they generated from the aforementioned Photovoice project. After identifying that the publicly available data did not include numerous hazards of concern to the watershed researchers and other Proctor Creek residents, the researchers decided that it was necessary to create their own database of indicators representing said hazards. 

### 2.4. Inclusion Criteria for Indicators

After brainstorming a list of potential indicators, some of which came from the aforementioned Proctor Creek Photovoice project, the watershed researchers adapted and agreed upon an indicator identification framework and inclusion criteria from the work of Badland et al. [45], which was initially undertaken to assess various livability indicators, including the natural environment, that likely contribute to health and well-being through the social determinants of health. Specifically, Badland et al.’s four inclusion criteria were adapted as a relevant approach to assess the aforementioned indicators for inclusion in our study: (1) Is the indicator significant to livability and/or the social determinants of health and well-being in urban areas? (2) Is the indicator specific and quantifiable? (3) Can the indicator be measured at the appropriate level(s) and scale(s) so that intra- and intercity comparisons be made? and (4) Is the indicator relevant to Atlanta urban planning policy? 

Once identified based on the aforementioned inclusion criteria, indicators were divided into three categories: (1) the indicator is promising because it meets at least 50% of the criteria; (2) the indicator may be useful but requires further development to meet the criteria; or (3) the indicator is not useful for our research purpose, either because it fails to meet the criteria of interest, or is redundant because of similar but more promising measures. Through a ranking process, the watershed researchers prioritized three locally relevant indicators: (1) locations where there is often standing water or where water commonly pools or collects; (2) locations where there is illegal dumping (in Proctor Creek or its tributaries or on land surfaces in the Proctor Creek Watershed); and (3) locations where there is faulty stormwater infrastructure (clogged or collapsed storm drains, sinkholes or depressions caused by inadequate drainage). 

### 2.5. Co-Development of a Digital Data Collection Mapping Tool

Through a collaborative process, the Proctor Creek Watershed researchers worked with faculty and students from the Georgia State University School of Public Health and Department of Geosciences to develop a digital data collection mapping tool using the Environmental Systems Research Institute (ESRI) ArcGIS Online program and the ArcGIS collector application (Redlands, CA, USA). This mapping tool, the Proctor Creek Citizen Science Application, is downloadable to smart phones and tablets and is connected to a database server that allows for real-time data collection, storage, and sharing. 

The app allows for the collection of photos and/or videos and prompts the user to record global positioning system (GPS) coordinates of the location being mapped. Data can be recorded by multiple app users simultaneously and updated on the server in real time, allowing teams of data recorders to physically see where other data collection is happening and to prevent duplication of efforts in the field. Use of the app is currently restricted to study participants, and the app is compatible with Apple and Android mobile phones and tablets. 

**Figure 1:**
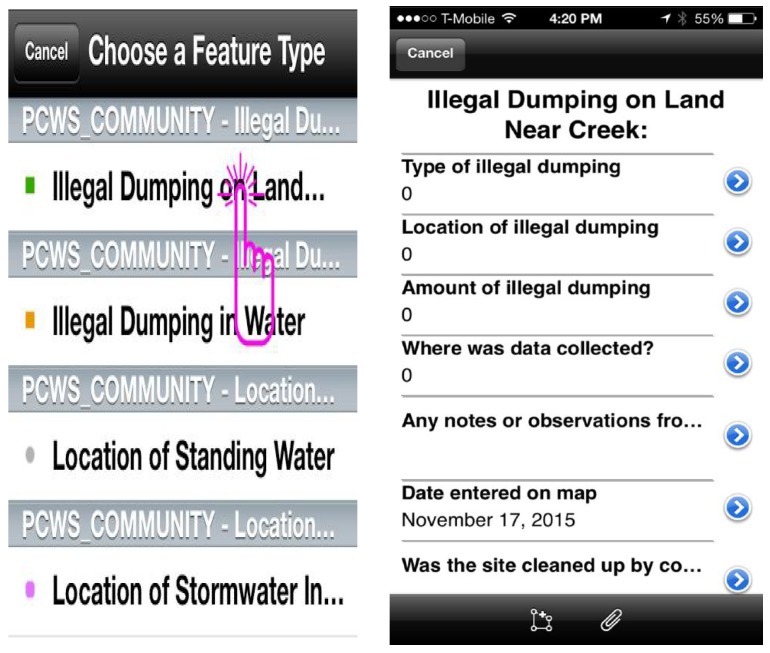
Examples of entry fields for data entry in the Proctor Creek Watershed Citizen Science App.

The Proctor Creek Watershed Researchers designed a series of data entry prompts for each of the indicators (hazards) that they prioritized for inclusion in the app. After meeting with the watershed researchers over several sessions to understand the type of data they wanted to collect with the app, the students collectively created a geodatabase of the study area including boundaries, parks, and neighborhood names within the watershed. The students also developed the series of data entry prompts into an easy-to-use, drop-down, multiple choice menu of data fields that correspond to each hazard identified and mapped (see Figure 1). The multiple choice format was chosen to standardize the type of data collected and to minimize data entry challenges for the end users entering data into the app. Data fields include type of hazard, location, amount, and other hazard-specific data as detailed in Table 1. Optional field notes from the user can also be entered into the database. 

Once the app was constructed by the students, they presented it to the watershed researchers who approved it and confirmed that all of their points of interest and desired capabilities had been addressed within it. None of the watershed researchers had any previous experience with mapping or in using mobile devices to collect data. Students conducted a 1 h training for watershed researchers on use of the app that included assistance in downloading and installing it to personal mobile devices, followed by practice using the associated drop-down menu, taking photos, and capturing GPS locations for sample hazards. Once all of the watershed researchers felt comfortable using the app, students and researchers divided into five groups to collect hazard data within the watershed boundaries. The students developed a “leave behind” set of step-by-step written and pictorial instructions for the researchers to refer back to for future use as needed. 

### 2.6. Data Collection

The 10 watershed researchers were paired with faculty and students from GSU in five field teams of four persons each to collect data within the Proctor Creek Watershed boundaries using the Proctor Creek Citizen Science App. Two watershed researchers were assigned to each team. The researchers determined the routes to travel for data collection based on their knowledge of areas that were heavily impacted by standing water, illegal dumping, and stormwater infrastructure challenges. The researchers began their mapping in two heavily impacted neighborhoods, English Avenue and Vine City, and moved further west into the lower reaches of the watershed. The routes corresponded with heavily traveled (by both car and foot traffic) arteries and the corresponding side streets.

Each team had a minimum of one device that was connected to the Proctor Creek Citizen Science App. Phase one of the data collection occurred during two separate 2 h field sessions in March and April 2015. Phase two of the data collection occurred during three subsequent sessions in May, June, and July 2015 and included only community researchers and the lead author. 

### 2.7. Data Analysis

The lead author and watershed researchers used ArcGIS Online functions (query and analysis tools) to aggregate and analyze data downloaded from the Proctor Creek Citizen Science App. A series of maps were generated to visually display the data collected by the research teams. Heat map analyses were conducted to visually explore density, and hot spot analyses were performed to map statistically significant patterns of clustering within the data. Selected results from this statistical analysis are included in the Results section. Each stressor was explored individually using the hot spot analysis tool, and a merged layer of related stressors exhibiting statistical significance were analyzed to produce a heat map. The queries conducted were determined by the watershed researchers, and results from executing them were shared and discussed with the watershed researchers for interpretation. The student collaborators developed a mapping manual for future use by the watershed researchers that details information on how to add data to the project from a desktop computer and how to conduct the aforementioned data analysis using ArcGIS Online. 

## 3. Results

**Figure 2:**
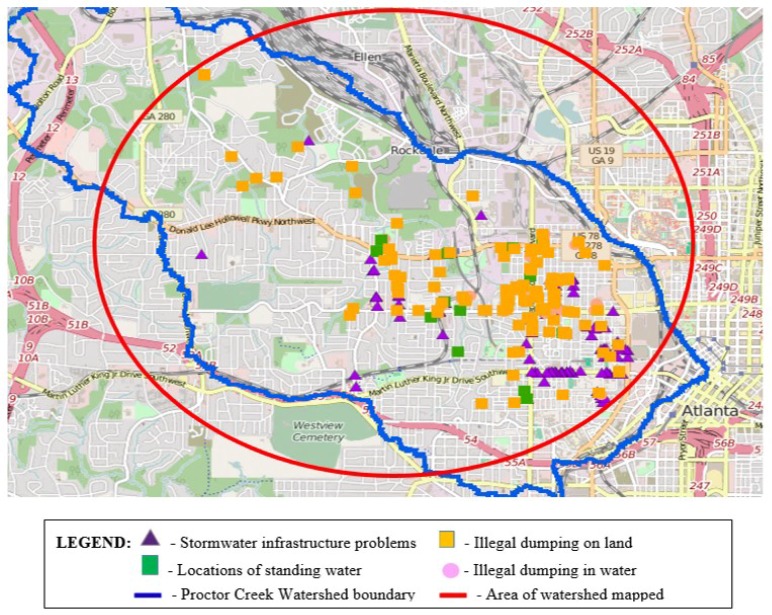
Community-generated map of Proctor Creek Hidden Hazards.

Over a period of 5 days (total 10 h), the community–university and community field teams mapped more than 50% of the watershed. We produced a community-generated map accompanied by a database that pinpoints exact locations of, and photographs/video representing, environmental hazards in the watershed. A total of 275 data points were generated across all indicators. Illegal dumping on land made up 44% (121 of 275) of the total data points followed by locations of stormwater infrastructure problems at 42% (116 of 275), locations with standing water at 9% (25 of 275), and illegal dumping in the creek at 4.7% (13 of 275). Point locations representing these hazards are displayed on the community-generated map (see Figure 2).

The data were analyzed using the ArcGIS Online hot spot spatial analysis tool to detect statistically significant hazard clusters using the Getis-Ord GI* statistic, a z-score returned for each feature in the dataset. The p-values and z-scores that result from this analysis help identify areas where high or low values cluster spatially. When considering statistically significant positive z-scores, the larger z-scores correspond to more intense clustering of high values (hot spots). Cold spots are identified when considering statistically significant negative z-scores. In this context, the smaller the z-score is, the more intense the clustering of low values [46]. 

**Figure 3:**
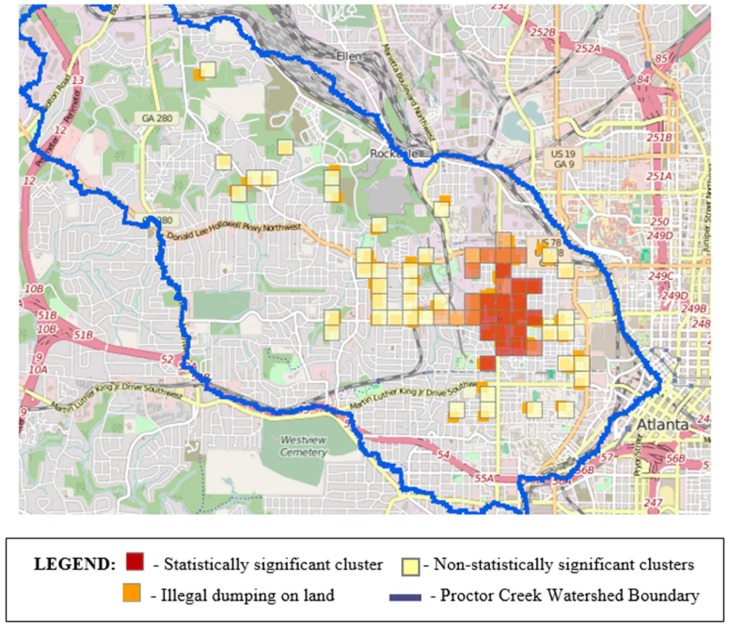
Statistically significant clustering of areas with illegal dumping mapped by community researchers in the Proctor Creek Watershed.

**Figure 4:**
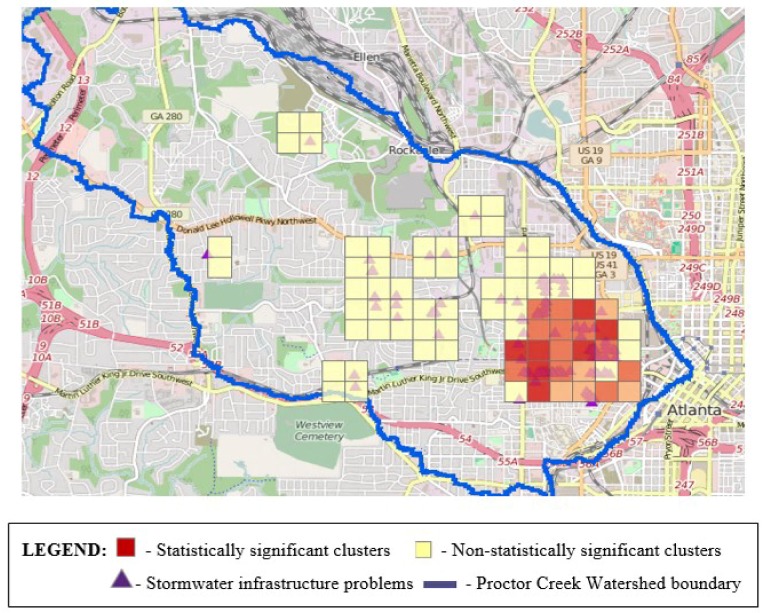
Statistically significant clustering of locations with stormwater infrastructure problems.

**Figure 5:**
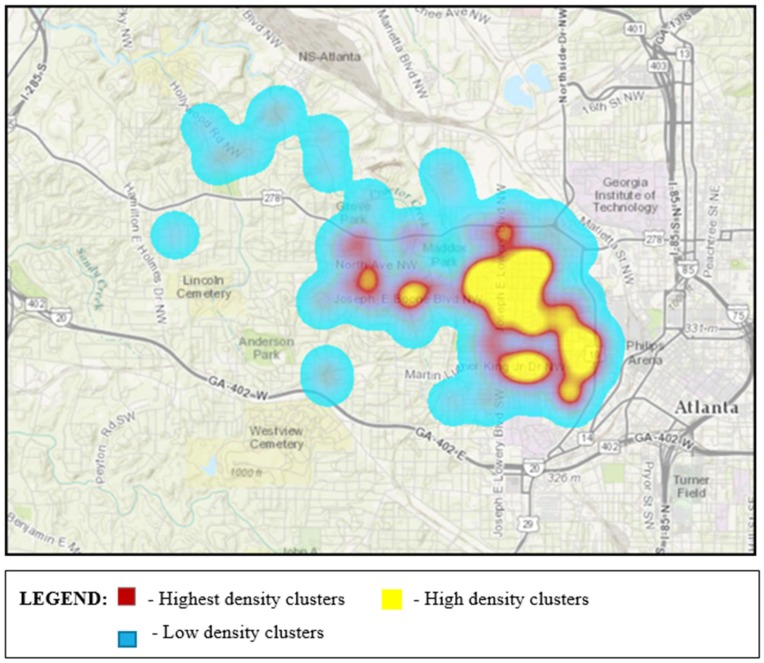
Areas of highest density (depicted by the colors yellow and red) of illegal dumping on land and locations of stormwater infrastructure problems in the Proctor Creek Watershed (does not denote statistical significance).

**Figure 6:**
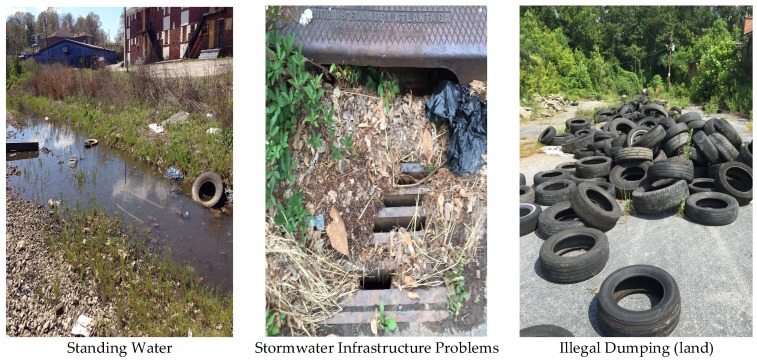
Examples of photographs documenting Proctor Creek Watershed Hazards.

In Figure 3 and Figure 4, the orange- and red-colored blocks represent hot spots or a statistically significant clustering of high values. The darker the color, the higher the confidence levels (ranging from 90% to 99%). Yellow blocks are not statistically significant. No cold spots (blue-colored blocks representing statistically significant clusters of low values) were identified in any of our analyses, however, there were both hot spots and areas in which the patterns are random (depicted by yellow blocks). Individual analyses of the illegal dumping on land and stormwater infrastructure challenges data revealed 28 and 23 statistically significant features, respectively, based on application of a false discovery rate (FDR) correction for multiple testing and spatial dependence. These clusters are shown in Figure 3 and Figure 4. In Figure 5, we display a heat map that allowed the community researchers to visually examine where the highest density of both illegal dumping on land and location of stormwater infrastructure problems exist. The map visually represents the largest areas where most of the points are concentrated. Because heat maps only account for the geographic location of point features on a map, statistical significance cannot be assumed [47]. To provide further evidence of environmental stressors, the mapping application also includes photographs taken by community researchers (examples provided in Figure 6).

## 4. Discussion

This study was designed to explore and document community knowledge of street-level environmental hazards. Unlike many GIS projects, the database design was controlled by the community research participants. Local knowledge and technical mapping expertise came together to enact a community plan that included collaborative design of the app, community-led data collection, and co-interpretation of the findings. The collaborative effort between community and university partners enabled a non-tech-savvy audience to effectively use technology to collect data about important community hazards, as informed by their lived experience and local community knowledge. This participatory mapping approach connected maps to visual stories of hazards that were “hidden in plain sight,”—abundant and widely distributed across parts of the Proctor Creek Watershed landscape, yet unaddressed and contributing to poor living conditions on Atlanta’s Westside.

Although mapping to date has not been conducted in the entire Proctor Creek Watershed, the field research teams identified statistically significant areas in the part mapped that warrant improvements with respect to illegally dumped trash and debris on land and in terms of the condition and maintenance of stormwater infrastructure (i.e., clogged and sometimes collapsed storm drains). Despite the existence of several non-statistically significant clusters, as shown in Figure 3 and Figure 4, the geographic areas that they represent are not devoid of pollution or other hazards that should be addressed.

The heat map generated by spatial analysis of merged data—representing illegal dumping on land and locations with stormwater infrastructure challenges—illuminated the need to pay attention to these areas. While heat maps are tools for data visualization, and the color gradients indicate areas of increasingly higher density (from blue to purple, red, orange, and yellow, respectively), these maps do not necessarily depict statistically significant data as do the maps displaying hot spots. Watershed researchers, however, found such maps useful in recent efforts to communicate to decision makers which areas in the watershed they deem necessary to prioritize for remedial action.

The data collected by community researchers, even in the initial stages, proved “community truths” through the validation of local, community knowledge with respect to the existence and location of, often overlooked, environmental hazards, especially ones not contained in publicly available datasets for the watershed. Proctor Creek Watershed residents are optimistic that having valid maps and spatial data accompanied with photographs can help provide an evidence base that will prompt remedial action by city officials and motivate fellow watershed residents to increase advocacy efforts designed to improve deleterious environmental conditions.

Maps speak the language of decision makers, and, in this case, the community-generated maps gave the community voice that was supported by location-specific visual evidence. They convey context about built environment stressors in the watershed and can ignite discourse about underlying root causes associated with community challenges. The results of this participatory mapping approach have helped community residents to create a place for themselves at planning, code enforcement, and watershed management decision-making tables. In addition to increasing community agency to press for remedial action and policy change, identification of hazard locations can also be used to plan community responses such as clean-ups and community education efforts to raise awareness about the causes of, consequences of, and solutions to illegal dumping and other challenges experienced in the Proctor Creek Watershed.

Despite its utility in helping to elevate and prioritize areas for greater public investment in community action, city services, and remedial measures, our methodological approach has several limitations. First, because the ArcGIS Collector application is primarily internet-based, there are occasional problems with accessing the platform for field data collection (though ArcGIS Collector offline data collection is possible with less data accuracy). It is also possible that some data points show up in the wrong place, requiring data to be validated. Use of the app requires internet-enabled computers and/or mobile devices and leaves out those without access to them. Although recent literature suggests that smartphones are beginning to bridge the digital divide because of wider accessibility, even in developing countries [48], smartphone users tend to be younger in age than general cell phone users [49,50], thereby adding a new dimension to the divide between those with and without access to contemporary communications devices. These younger users, when coming from low-income households, are more burdened by costs associated with accessing the internet from mobile devices than from traditional computing platforms [51]. Using apps like the one described herein may then prove costly if mobile users have a limited data plan. Furthermore, while access to the ArcGIS Online platform—on which the app operates—is free, a subscription is required to perform data analysis (though, in our case, community researchers have access to the analysis options through a university account). 

Our data did not represent findings from across the entire watershed and are limited by the choices community researchers made about where to collect data based on their local knowledge of environmental stressors. Meaningful data analysis was subsequently limited due to its dependence on a minimum number of points to identify statistically significant spatial clustering within specific hazard types. Mapping the entire watershed may have revealed additional areas of statistical significance; also, the content that we designed the Proctor Creek Citizen Science App to collect was not streamlined to allow for greatest utility in advanced data analyses. For example, while the app prompts users to quantify the amount of illegal dumping identified, it does not do so for the amount of standing water nor does it prompt users to distinguish highly clogged storm drains from those that are minimally clogged. In the future, being able to identify the data points with the highest impacts through data analysis queries will enhance the ability of this approach to help planners and other municipal officials determine which areas are most in need of remediation.

Also, as a project driven largely by community (watershed) researchers, user subjectivity can also influence what is deemed significant and consequently what should be documented. Our app allows users to document visual evidence to substantiate the data points collected, however, the decision to map or not to map lies in the hands of individual researchers. Although there was agreement on the environmental stressors to document in the study area, there were differing perspectives with respect to mapping specific sites. For instance, because of the seemingly ubiquitous nature of illegal dumping in the Proctor Creek Watershed, this hazard was underrepresented in the community-generated data. In some cases, watershed researchers felt that occurrences of illegal dumping were so commonplace that every pile did not rise to the level of needing to be mapped. Future efforts should address the potential bias and user subjectivity associated with mapping. In the aforementioned case of illegal dumping, the impacted sites that researchers chose not to map may be just as important as those that they did map.

### Directions for Action and Future Research

There is consensus among the watershed researchers that maps, photographic documentation, and GPS coordinates are vital to having productive interaction with government officials to advocate for advancements in corrective action and remediation. Through this collaborative mapping application, Proctor Creek Watershed residents now have a mechanism with which they can use to tell their story using spatial data that is difficult for government officials to ignore. If repeated in the designated study area over time, such an approach can also be used to track remedial action and spatiotemporal changes in urban living conditions. Highlighting otherwise hidden hazards is the first step in ensuring that the community’s local knowledge receives the attention it deserves. At minimum, the utility of this approach for local planning, watershed management, and code enforcement practices can be enhanced as additional data is collected and analyzed. This approach can also be enhanced by mapping street-level community assets that promote and protect health. It will be important to determine if the results of such a participatory mapping approach, that includes both assets and hazards, can lead to the production of a comprehensive, fine-grained data layer that is appropriate to integrate with publicly available data and demographic data for the purposes of cumulative risk assessment and impact analyses. In contrast to the environmental hazards in the Proctor Creek Watershed identified in other datasets, none of the relevant environmental hazards identified by the watershed researchers were chemical hazards. Uncovering these hidden hazards for integration with publicly available hazard data is consistent with other community-engaged research to explore nonchemical stressors in the context of approaches like cumulative risk assessment. The integration of publicly available data with data collected through participatory mapping will lead to more accurate maps of watershed residents’ proximity to hazard sources than can be generated with publicly available data alone. 

Conducting training refresher sessions to help the watershed researchers retain app user “know-how” and providing training to expand use of the app to additional residents will help to sustain ongoing engagement. Presentation of preliminary data collected with the app has already led to discussions with city government officials about identification of scrap tires for which funds can be obtained for clean-up from a state government program. The aforementioned group of researchers have now also been trained to identify illicit discharges (pollution from pipes and drains) into Proctor Creek—data that can be added to enhance the preexisting app. A relationship with city watershed protection officials is being forged that is expected to yield faster responses to watershed-based problems than prior to these community residents’ engagement in this process. 

## 5. Conclusions

This study contributes to ongoing discourse with respect to meaningful citizen engagement in urban planning and health promotion strategies to improve built environment outcomes. Our approach is consistent with environmental justice principles and recognized methods for effective and authentic public participation in environmental and other public health decision making. The study demonstrates the benefits derived from using community-generated spatial data to examine community concerns. Following such an approach can help influence and democratize decision making by putting data in the hands of community residents to help prioritize and leverage action when issues go unseen or are consistently unaddressed. 

ArcGIS Online and ArcGIS Collector are open and customizable platforms. Both can be adapted to meet specialized needs and concerns in a wide range of locales. Because of the resources required, however, decisions to use these tools should be pursued on a case-by-case basis and should not be considered universal solutions. Nevertheless, community-based mapping applications offer viable options for activities that expand meaningful community engagement alternatives for those desiring to influence local, urban governance. When community-based organizations partner in meaningful ways with academic institutions, resource limitations can be overcome: both in terms of access to devices needed to conduct field activities, as well as the technical expertise required to design digital data collection tools based on needs expressed by community stakeholders. Through successful collaboration with and engagement of impacted residents, local community knowledge of street-level environmental hazards can be elevated and data gaps filled. Exposing such “hidden hazards” through collecting and analyzing data about them can advance environmental justice agendas, improve local area environmental health analyses, and help initiate corrective action.

## Figures and Tables

**Table 1:** Hazard-specific data choices from drop-down menus collected in the Proctor Creek Watershed Citizen Science App.

Type of Hazard Recorded in App	Hazard-Specific Information Recorded in App
**Standing Water/Pooling Water**	- Raining right now - Not raining right now - Not raining now, but rained in the last 48 h - Visible evidence of mold on buildings nearby - Presence of damp, moldy smell in the area
**Type of Illegal Dumping in Water**	- Sewage/floatable solid - Non-point source pollution (bottles, cans, potato chip bags, etc.) - Heavy debris (tires, heavy items that someone most likely had to put directly into the creek) - Other
**Type of Illegal Dumping on Land**	- Construction or other building materials - Scrap tires - Housing debris (couches, mattresses, furniture, etc.) - Assorted debris (mixture of household trash, litter, cans, bottles, plastic bags, etc.) - Other
**Type of Stormwater Infrastructure Problems**	- Clogged storm drains - Clogged stormwater pipes - Collapsed storm drains - Sinkholes/depressions
